# CLCA2 is a positive regulator of store-operated calcium entry and TMEM16A

**DOI:** 10.1371/journal.pone.0196512

**Published:** 2018-05-14

**Authors:** Aarushi Sharma, Grace Ramena, Yufang Yin, Louis Premkumar, Randolph C. Elble

**Affiliations:** 1 Department of Pharmacology, Southern Illinois University School of Medicine, Springfield, IL, United States of America; 2 Medical Microbiology, Immunology and Cell Biology, Southern Illinois University School of Medicine, Springfield, IL, United States of America; 3 Simmons Cancer Institute, Southern Illinois University School of Medicine, Springfield, IL, United States of America; University of Louisville, UNITED STATES

## Abstract

The Chloride Channel Accessory (CLCA) protein family was first characterized as regulators of calcium-activated chloride channel (CaCC) currents (I_CaCC_), but the mechanism has not been fully established. We hypothesized that CLCAs might regulate I_CaCC_ by modulating intracellular calcium levels. In cells stably expressing human CLCA2 or vector, we found by calcium imaging that CLCA2 moderately enhanced intracellular-store release but dramatically increased store-operated entry of calcium upon cytosolic depletion. Moreover, another family member, CLCA1, produced similar effects on intracellular calcium mobilization. Co-immunoprecipitation revealed that CLCA2 interacted with the plasma membrane store-operated calcium channel ORAI-1 and the ER calcium sensor STIM-1. The effect of CLCA2 on I_CaCC_ was tested in HEK293 stably expressing calcium-activated chloride channel TMEM16A. Co-expression of CLCA2 nearly doubled I_CaCC_ in response to a calcium ionophore. These results unveil a new mechanism by which CLCA family members activate I_CaCC_ and suggest a broader role in calcium-dependent processes.

## 1. Introduction

Calcium-activated chloride channels play an essential role in the physiology of many cell types. In epithelial cells, they drive transepithelial secretion of fluids and mucus in response to cytokines such as IL-13 [**1,2**]. In smooth muscle, I_CaCC_ mediates contraction in response to signaling molecules such as histamine, norepinephrine, and endothelin that stimulate release of intracellular calcium [**3**].

Despite their obvious physiological significance, the molecular identity of CaCCs was discovered only recently. Two members of the Anoctamin family of multipass membrane proteins, TMEM16A and TMEM16B, were found to mediate a current with the same properties as the classical I_CaCC_ [**4, 5, 6, 7**]. While TMEM16B is chiefly expressed in the central nervous system and implicated in olfactory transduction, TMEM16A is widely expressed in epithelia and other cell types in which I_CaCC_ had previously been characterized [**7, 8**]. Subsequently, genetic and physiological evidence has accumulated for TMEM16A roles in glandular secretion; expression of fluids and mucus; smooth muscle contraction in airway, gut, and vasculature; and sensory transduction of heat and pain [**9, 3**]. TMEM16A also plays a pivotal role in related pathologies such as asthma, diabetes, and hypertension [**9, 10, 11, 12, 13**].

The activation of TMEM16A-mediated current by calcium is now well established. One mode is by calcium release from the ER via the inositol 1,4,5-trisphosphate receptor (IP3R), a ligand-dependent calcium channel that associates with TMEM16A at the plasma membrane [**3, 8**]. The ligand IP3 is generated by phospholipase C (PLC) in response to binding of extracellular signaling molecules to PLC-beta-linked G-protein-coupled receptors and PLC-gamma-linked receptor tyrosine kinases [**14, 15, 16**]. Exhaustion of ER calcium stores by IP3R-mediated calcium release is detected by a sensor in the ER membrane, STIM-1; STIM-1 becomes phosphorylated, allowing it to associate with and activate a plasma membrane calcium channel termed ORAI [**17, 18, 19**]. ORAI admits extracellular calcium into the cytosol in a process called store-operated calcium entry (SOCE), and ER calcium is then replenished by calcium pumps in the ER membrane termed SERCA [**20, 21**]. Thus, SOCE allows further stimulation of TMEM16A-mediated I_CaCC_ by renewing ER calcium [**3**]. The dependence of this channel on SOCE was recently demonstrated in humans with deficient sweat expression; the dysfunction arises from mutations in ORAI-1 that reduce TMEM16A activity [**22**].

All CLCA family members tested by ectopic expression have been found to enhance calcium-activated chloride currents, and CLCA proteins were initially thought to be channel subunits [**23, 24, 25**]. However, it was later determined that their transmembrane topology was incompatible with that function and they instead constituted a new family of self-cleaving metalloproteases [**26, 27, 28**]. It was therefore surmised that CLCAs must instead activate an unknown endogenous CaCC. Accordingly, Hamann et al. (2009) [**29**] later demonstrated that ectopic expression of CLCA1 in HEK293 cells did indeed enhance the amplitude of such a channel current. The channel responsible was recently identified as TMEM16A [**30**].

Like TMEM16A, CLCA1 has been found to play a role in asthma, cystic fibrosis, and other inflammatory pathologies of airways [**31**, **32**, **33**]. CLCA2 on the other hand is better known for its role in cancer. This gene is induced by p53 in response to cell stress, plays an essential role in epithelial differentiation, and is frequently downregulated during progression of breast, prostate, and other adenocarcinomas [**34, 35, 36, 37**]. In addition, different mutations of CLCA2 have been linked to inflammatory bowel disease, familial cardiac disease, and chronic lymphocytic leukemia [**38, 39, 40, 41**].

Whether CLCA1 and CLCA2 are functionally redundant remains largely unanswered. Although their domain structure is similar, their amino acid conservation is only about 40%, and CLCA2 has a C-terminal transmembrane segment, while CLCA1 is fully secreted [**27, 28**].

CLCA1 was recently reported to enhance the activity of TMEM16A by direct interaction at the plasma membrane [**30**]. We report here that CLCA2 also activates TMEM16A-dependent chloride current but by a different mechanism. Instead of physically interacting with TMEM16A, we found that CLCA2 enhanced intracellular calcium stores and SOCE. Furthermore, CLCA1 had similar effects on calcium mobilization. Immunoprecipitation experiments revealed that CLCA2 interacted with two key mediators of SOCE, STIM-1 and ORAI-1.

These results suggest that CLCA proteins regulate I_CaCC_ by more than one mechanism. They further suggest that the conserved function of CLCA proteins is to regulate cytosolic calcium levels in response to multiple stimuli. This discovery may be relevant not only to physiological processes that are regulated by I_CaCC_ but to any event that is dependent on cytosolic calcium, including differentiation and apoptosis.

## 2. Materials and methods

### 2.1 Reagents

Ionomycin was purchased from Adipogen; Cyclopiazonic Acid (CPA) was obtained from Tocris; Pluronic-F12 from Biotium and Life Technologies; Fluo4-AM from Life Technologies; BTP-2 from EMD-Millipore; 4,4'-diisothiocyano-2,2'-stilbenedisulfonic acid (DIDS) from Sigma.

### 2.2 Antibodies

Anti-FLAG mouse monoclonal antibody M2 was obtained from Stratagene or Sigma. CLCA2 was detected by TVE20 rabbit polyclonal affinity-purified antibody [**28**] or Sigma Prestige antibody #HPA47192. TMEM16A mAb C5 and beta-tubulin mAb were from Santa Cruz. Anti-Myc mAb was prepared from 9E10 hybridoma cell line (ATCC). ORAI-1 and STIM-1 rabbit pAb were obtained from ProteinTech Group. Primary antibodies were used at 1:1000 for immunoblots and 1:100 for IF. Streptavidin-IR680 and goat-anti-rabbit and anti-mouse tagged with IR800 were obtained from LICOR. Goat-anti-mouse 649-Dylight was from Thermo Scientific. Secondary antibodies were used at 1:20,000 for immunoblots and 1:1000 for IF.

### 2.3 Cell culture

HEK293 cells (ATCC) were grown in Dulbecco’s modified Eagle’s medium containing 10% FBS at 37^o^ Celsius and 5% CO2. Cells were validated by IHC for adenoviral E1A. MCF10A cells were obtained from ATCC in 2011 and frozen at low passage. MCF10A and its knockdown derivative were grown in CM as described [**35**]. Expression of shRNA1 targeting CLCA2 was induced with doxycycline as described [**36**]. Mycoplasma testing was conducted by PCR (ATCC).

### 2.4 Lentiviral methods

To obtain stable HEK293 transductants expressing TMEM16A, TMEM16A-Myc lacking the Flag tag was obtained from TMEM16A-Myc-DDKpCMV6Entry (Origene) and inserted into pLenti-GFP-Hygro in place of the GFP [**42**]. Packaging and infection were performed as described [**36**]. Transduced cells were selected with 50 micrograms/ml hygromycin. HEK293 cells expressing CLCA2 or CLCA1 were obtained by transduction of pLex clones (OpenBiosystems). Transduced cells were selected with 0.5 to 1 micrograms/ml puromycin for at least two weeks.

### 2.5 Protein and immunomethods

For protein analysis, cleared NP40 lysates were prepared from 10-cm dishes containing cells at 100% confluency as described in 25mM Tris pH 7.6, 150mM NaCl, 2.5mM MgCl2, 0.5mM EDTA, 0.5% NP-40, 1mM DTT, 5% glycerol, 1% aprotinin [**36**]. Protein concentration in lysates was measured by BCA assay, and 50 micrograms of protein was loaded per lane. Immunoprecipitations were performed as described, except that protein A/G beads were washed a total of four times before eluting proteins [**28**]. The protein size marker was Dual color (Bio-Rad). Immunoblots were processed as described [**36**]. In general, primary antibodies were diluted 1:1000 and fluorescent secondary antibodies at 1:20,000. Protein expression was quantified on an Odyssey instrument (Licor). For chemical cross-linking of proteins, cells were treated with the membrane-permeable crosslinking agent, DSS (disuccinimidyl suberate, dissolved in DMSO; Pierce) for 25 min at room temperature before quenching and collecting cell lysates [**28**].

### 2.6 Surface biotinylation

Cells in culture dishes were cooled on ice to prevent endocytosis before treatment with 0.1mg/ml of LHS-SS-long arm biotin (Pierce) for 25min as described [**28**]. Briefly, the reaction was quenched by washing with 50mM NH4Cl, 0.1% BSA in PBS with Ca2+ and Mg2+, and cell lysates were collected. To confirm surface restriction of labeling, avidin beads (Pierce) were used to collect biotinylated proteins for immunoblot with anti-tubulin antibody.

### 2.7 Calcium imaging

Calcium was imaged by loading cells with Fluo4-AcetoxyMethyl (Fluo4-AM) ester (Molecular Probes). Fluo-4AM stock solution (0.5mM) was prepared by dissolving in DMSO and Pluronic F127 dispersing agent (20% v/v stock solution in DMSO). Cells were loaded with 3 micromolar Fluo-4AM in HBSS or extracellular buffer without calcium at 37°C in reduced light. After incubation, the glass coverslip with dye-loaded cells was transferred to extracellular buffer without calcium to record changes in fluorescence intensity. Basal fluorescence was recorded for 2min. Cells were then treated with ionomycin (2 micromolar final concentration dissolved in extracellular buffer without calcium) for 4 minutes followed by 2mM extracellular calcium to determine the difference in calcium entry between cell lines. To measure SOCE, cells were treated with CPA (cyclopiazonic acid; 15–20 micromolar final concentration) in the absence of calcium for 25 minutes followed by 2mM extracellular calcium addition. *Instrumentation*: Fluo-4 was excited at 488 nm, and emitted fluorescence was filtered with a 535±25 nm band pass filter on a Leica DMEIRE2 microscope (Plymouth, MN) equipped with Lambda DG-4 illumination system (Sutter Instruments, Novato, CA) and Retiga Ex (QImaging, Surrey, BC, Canada). Data were processed using IP Lab3.7 software (Scanolytics, Fairfax, VA). After subtracting basal fluorescence from all readings, the change in fluorescence was plotted to represent the change in intracellular Ca^2+^ levels. The total number of cells individually measured per experiment was *n*, and experiments were repeated 2–3 times.

### 2.8 Immunofluorescence and confocal analysis

HEK293 cells stably transduced with CLCA2-Flag were grown on poly-L-lysine coated coverslips. The cells were fixed with cold methanol and blocked with blocking buffer (1% FBS, 1% BSA in PBS) overnight at 4°C. Cells were incubated 4h with antibodies for STIM-1 (1:100) or Flag (1:1000) in blocking buffer, followed by secondary antibodies labeled with AlexaFluor 568 or AlexaFluor 488 (Invitrogen) according to manufacturers’ recommendations. Nuclei were stained with Hoechst. Coverslips were mounted onto slides using ProLong Gold mounting medium (Invitrogen) for confocal microscopy (Leica SP5). Z-stack images were collected using a 1μm step size and optical cross-sections. Images were obtained using the Leica LAS AF Lite software. TMEM16A images were collected using an Olympus BX41 compound fluorescent microscope with a 40x objective.

### 2.9 Whole-cell patch clamp

HEK293 cells and transduced sublines were seeded onto poly-L-lysine coated coverslips and patch-clamped within 24h. The extracellular solution contained (in mM) NaCl 126, HEPES 10, MgCl2 2, sucrose 28, CaCl2 2; pH adjusted to 7.4 with Tris-HCl buffer. The intracellular solution was (in mM) NMDG 126, HEPES 10, MgCl2 2, sucrose 30, CaCl2 0.366, EGTA 1, Mg-ATP 5; pH adjusted to 7.4 with Tris-HCl buffer. The final chloride concentrations of the extracellular and intracellular buffers were 133 and 99mM, respectively, measured using a pHOx Ultra analyzer (Nova Biomedicals). The solutions were equal in osmolarity, 300+/-5mOsM. Cells were held at -60 mV and currents were recorded in response to a ramp protocol (from -60 to +60 mV) using an Axopatch 200B integrating patch-clamp amplifier (Molecular Devices, LLC. Sunnyvale, CA). All experiments were conducted at room temperature. Data were digitized (VR-10B; InstruTech, Great Neck, NY) and stored on a computer using a LabView interface (National Instruments). For analysis, data were filtered at 2.5 kHz (–3 dB frequency with an eight-pole low-pass Bessel filter; LPF-8; Warner Instruments) and digitized at 5 kHz [**36**]. Initial experiments revealed that capacitance between HEK293 cells varied by less than 10%. Therefore all currents were expressed as pA, and capacitance was not routinely measured.

### 2.10 Statistics

Statistical analysis was performed using GraphPad Prism 5.0. All pairwise comparisons were tested for significance using Student’s t-test, and p-values < 0.05 were considered significant (*). p-values < 0.01 were flagged with two stars (**), and p-values < 0.001 were flagged with three stars (***). Error bars represent mean +/- SEM. The numbers of samples and experimental repetitions are indicated in the figure legends.

## 3. Results

### 3.1 CLCA2 enhances store-operated calcium entry

**Fig 1:**
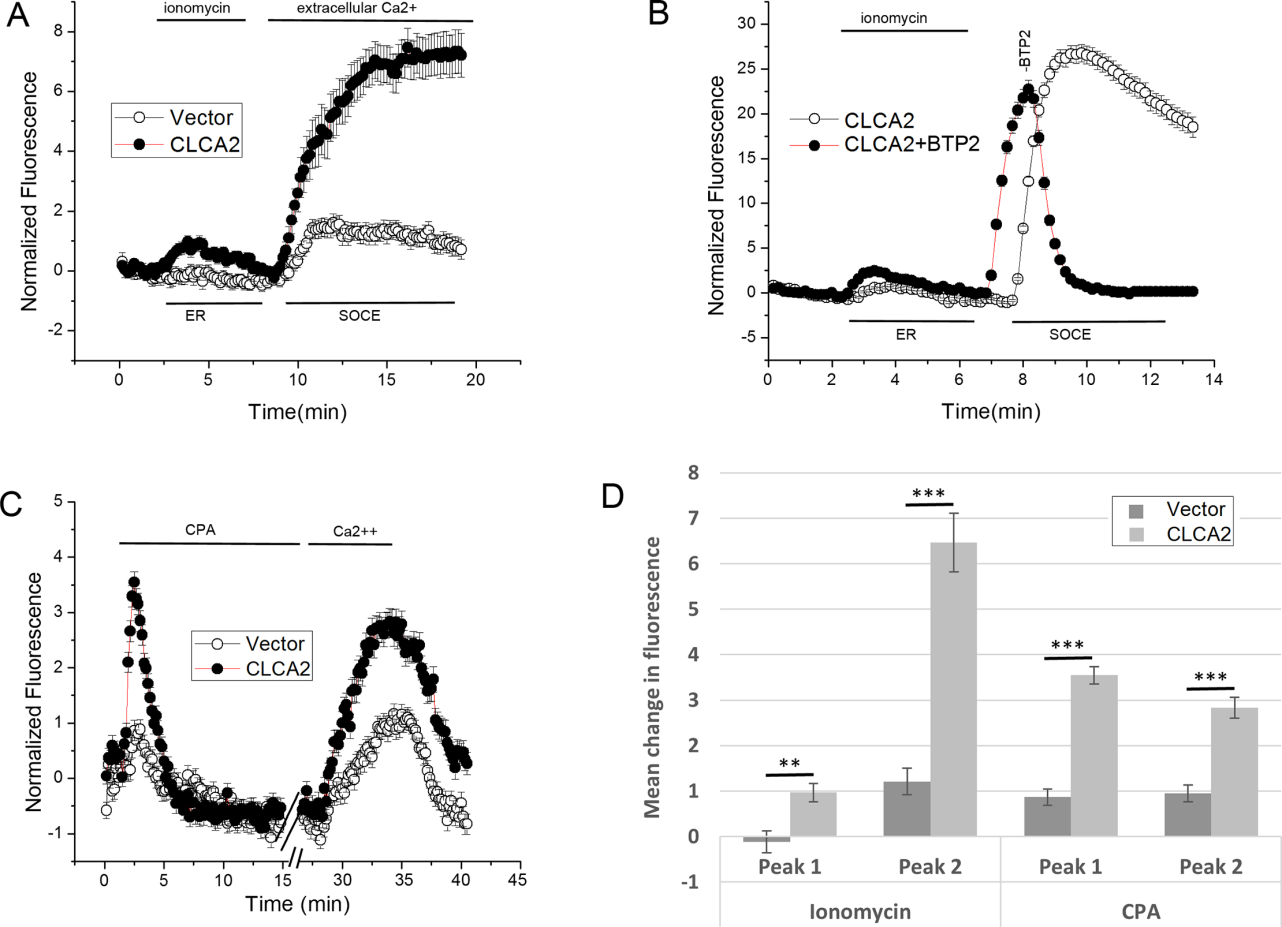
CLCA2 enhances ER calcium stores and SOCE. Calcium imaging studies using HEK293 cells loaded with calcium fluorophore Fluo-4. (A) Cells were treated with 2 micromolar ionomycin in the absence of extracellular calcium to allow ER depletion, then calcium was added to allow SOCE. For CLCA2, n = 52; for control, n = 49. (B) CLCA2-expressing cells were treated with ionomycin, and the SOCE inhibitor BTP-2 (10 micromolar) was added after extracellular calcium (n = 121). Control cells expressed CLCA2 but were untreated with BTP-2 (n = 124). The temporal displacement in the SOCE peaks was due to a minor difference in timing of calcium addition. (C) The same cell lines were treated with SERCA inhibitor CPA (15 micromolar). For CLCA2, n = 246; for control, n = 240. (D) Bar graph showing enhancement by CLCA2 of cytosolic calcium from the ER (Peak 1) and SOCE (Peak 2) in A and C. The height of each peak minus background is plotted. P-values were determined for pairwise comparisons between CLCA2 and control peaks. For ionomycin, Peak 1 p = 0.001, and Peak 2 p = 4.58x10^-11^. For CPA, Peak 1, p = 2.63 x10^-21^, and Peak 2 p = 9.23 x10^-10^. For A and C, traces represent the mean of three independent experiments.

To test whether CLCA2 affects intracellular calcium trafficking, we performed calcium-imaging studies using the fluorescent calcium indicator Fluo-4 in HEK293 cells transduced with CLCA2 or vector. Cells were treated with 2 micromolar ionomycin in the absence of extracellular calcium to allow exhaustion of intracellular stores and activation of store-operated calcium channels in the plasma membrane. Ionomycin is a calcium ionophore that initially causes release of calcium from the ER but later allows influx of extracellular calcium [**43**]. Upon restoration of extracellular calcium at 400 seconds, we observed a robust and dramatic increase in calcium entry in cells expressing CLCA2 compared to control cells (Fig 1A, SOCE; 1D, Peak 2). These cells also exhibited an earlier increase in intracellular calcium at 200 seconds, suggesting that CLCA2 might enhance calcium-charging of the ER in addition to enhancing SOCE (Fig 1A, ER; 1D, Peak 1).

To confirm that the second peak represented SOCE, the experiment was repeated using an inhibitor of SOCE, BTP-2 [**44**]. Control cells expressed CLCA2 but were untreated with BTP-2. Addition of BTP-2 after extracellular calcium triggered an immediate, steep drop in cytosolic calcium, indicating that calcium influx was due to SOCE (Fig 1B). The amplitude of the SOCE peak was higher than in 1A. This may be a consequence of variability in expression of the CLCA2 transgene with passage number.

To further confirm these results, we used a SERCA-pump inhibitor, cyclopiazonic acid (CPA). SERCA-pumps are responsible for sequestering cytosolic calcium in the ER. Inhibitors allow calcium release and prevent refilling. The depletion of calcium from the ER and cytosol activates SOCE [**17, 20**]. Accordingly, cells preloaded with Fluo-4 were treated with CPA for 30 minutes in the absence of extracellular calcium, followed by a 5 minute exposure to 2mM extracellular calcium, then a wash to bring the fluorescence back to baseline. Calcium influx was more robust in cells expressing CLCA2 (Fig 1C and 1D, confirming that CLCA2 enhances SOCE and suggesting this as a mechanism for regulating I_CaCC_. Moreover, we also observed more calcium release from the ER in response to CPA (Fig 1C and 1D), suggesting that CLCA2 promotes calcium storage in the ER.

### 3.2 CLCA2 knockdown reduces intracellular calcium stores and SOCE in MCF10A

**Fig 2:**
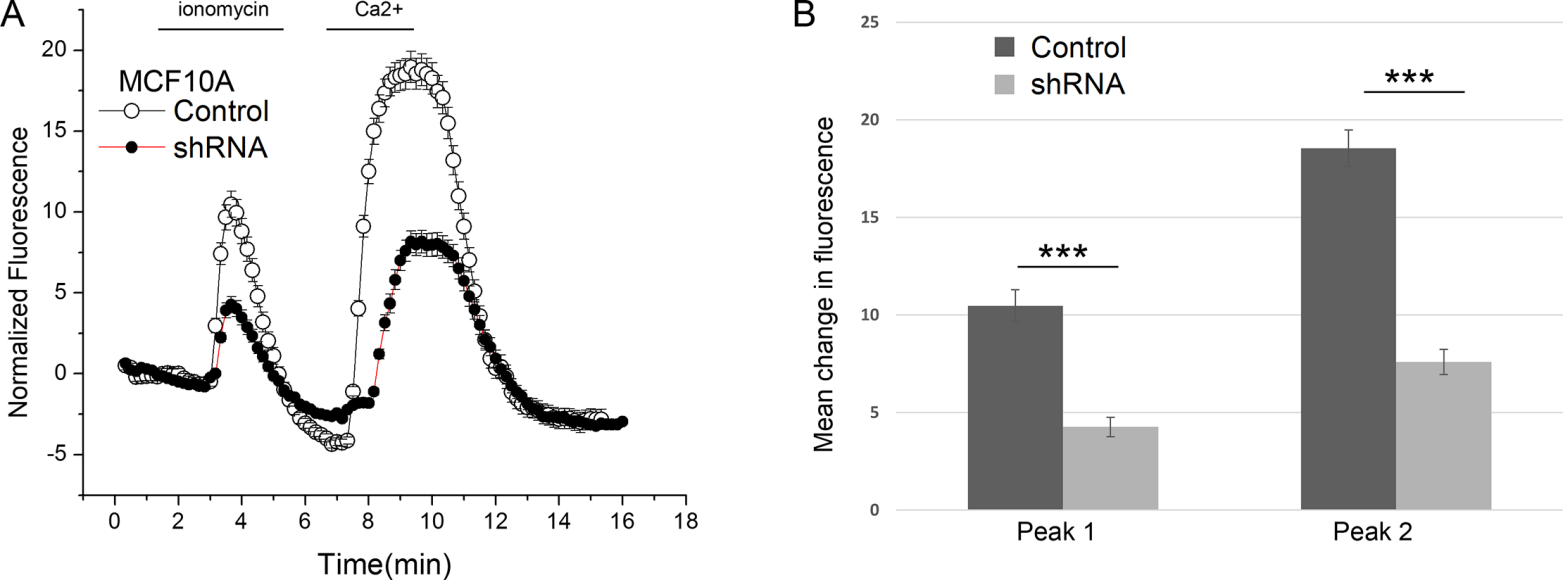
Effect of CLCA2 knockdown on calcium trafficking. (A) Calcium imaging in MCF10A mammary epithelial cells with CLCA2 knockdown (shRNA; n = 134) or vector (control; n = 196). Cells were treated with ionomycin as in Fig 1 except that calcium was withdrawn after appearance of Peak 2 as indicated by bars at top. (B) The height of each peak minus background is plotted. Peak 1, p = 1.77 x10^-9^. Peak 2, p = 6.10 x10^-18^.

To determine the importance of CLCA2 in calcium trafficking in a cell that normally expresses it, we knocked down its expression in MCF10A immortalized mammary epithelial cells. We had previously established the efficacy of the shRNA construct in attenuating CLCA2 expression in these cells ([**36]**). We observed that MCF10A cells exhibited greater release of intracellular calcium and greater SOCE than HEK293 (Fig 2). Attenuation of CLCA2 substantially reduced the amplitude of both peaks. (Note that the absolute peak amplitudes should not be compared between cell types because of possible differences in expression levels of STIM1, ORAI1, and other factors in addition to CLCA2 that could affect calcium storage and trafficking.)

### 3.3 CLCA1 also enhances calcium release

**Fig 3:**
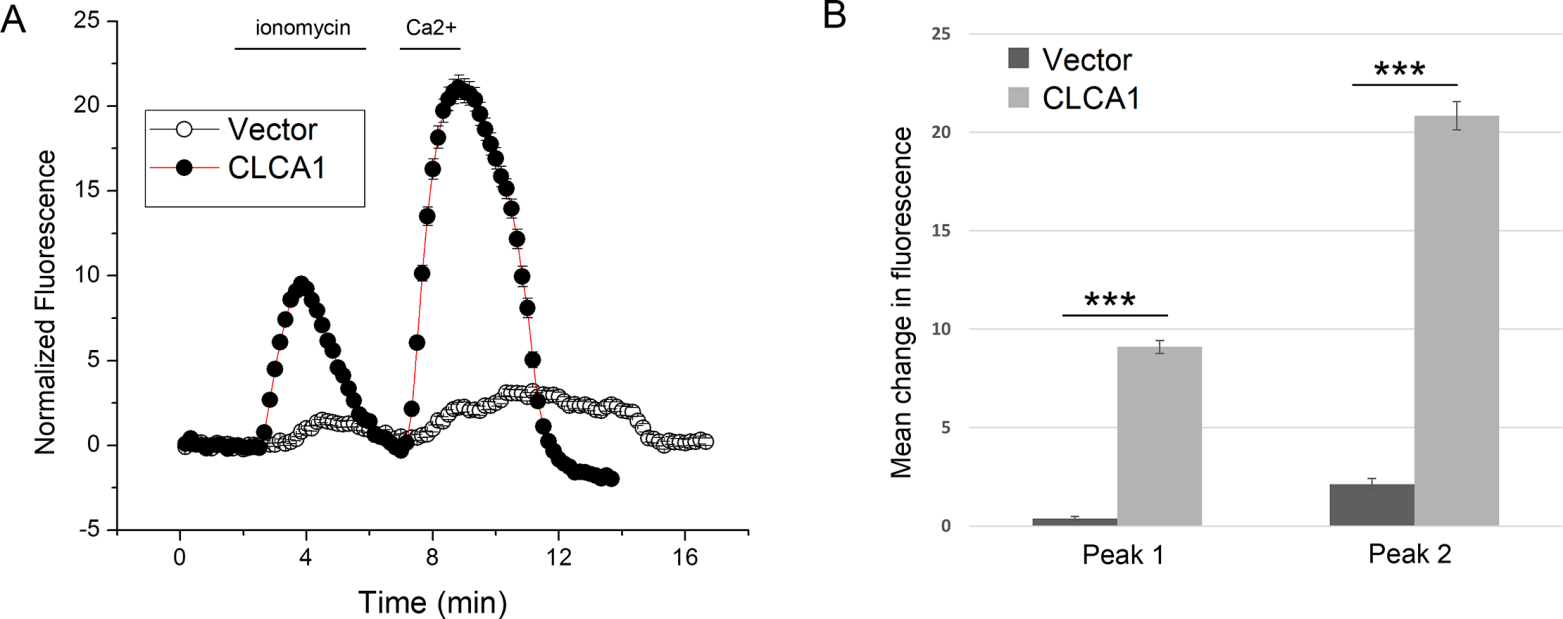
A second family member, CLCA1, also enhances ER calcium release and SOCE. (A) Cells stably expressing CLCA1 (n = 228 cells) or vector (n = 219) and loaded with Fluo-4 were treated with ionomycin and cytosolic calcium was measured over time as in Fig 2. (B) Amplitudes of peaks 1 and 2 were plotted. For pairwise comparisons between CLCA1 and vector, the p-value of Peak 1 was 3.2 x10^-87^ and the p-value of Peak 2 was 5.07 x10^-84^.

The CLCA2 ectodomain is conserved throughout the CLCA family, suggesting that other CLCA family members might have the same ability to modulate intracellular calcium levels. CLCA1 was recently shown to modulate TMEM16A conductance, apparently by interaction between the TMEM16A ectodomain and CLCA1 at the cell surface [**30**]. Here, using the same protocol as in Fig 2, we tested whether CLCA1 might also modulate intracellular calcium. We observed that HEK293 cells stably expressing CLCA1 had very similar effects on calcium storage and entry to those expressing CLCA2 (Fig 3).

### 3.4 CLCA2 interacts with STIM-1 and ORAI-1

**Fig 4:**
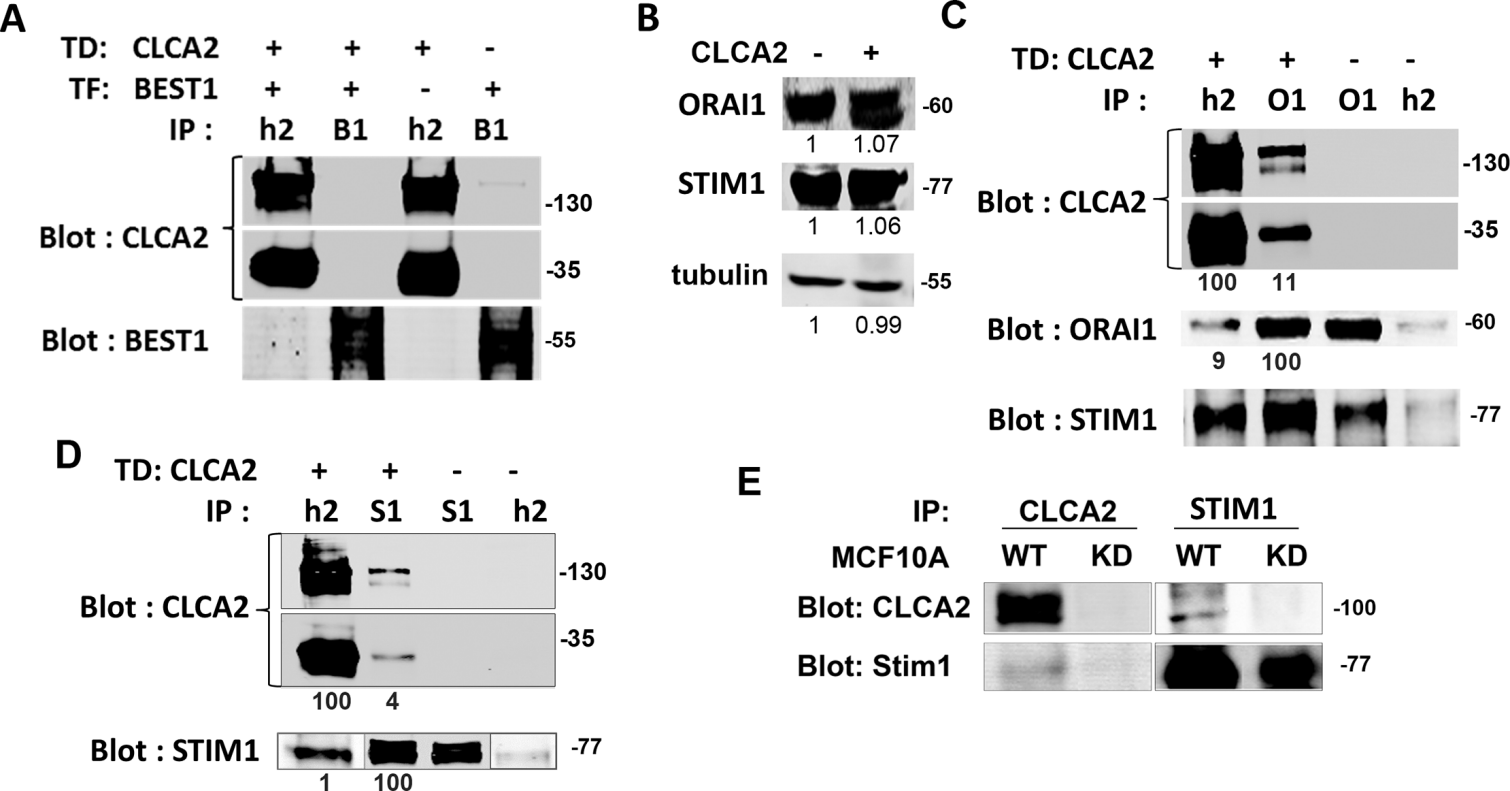
CLCA2 forms a stable complex with STIM-1 calcium sensor and ORAI-1 calcium channel but not with Best1. (A) Immunoprecipitations and immunoblots from HEK293 cells transduced with CLCA2-flag or vector. CLCA2 (h2) and Bestrophin1 antibodies (B1) failed to precipitate the other protein. (B) Immunoblot showing equal expression of STIM-1 and ORAI-1 irrespective of ectopic CLCA2 in HEK293. (C) Co-immunoprecipitation of CLCA2 and ORAI-1. The percentage of co-precipitation compared to immunoprecipitation is indicated below each band after subtracting non-specific background. Signal intensities were determined by fluorimetric scans (Licor Odyssey). O1, ORAI-1 antibody; S1, STIM-1 antibody. (D) Co-immunoprecipitation of CLCA2 and STIM-1. Lanes 2 and 3 of the STIM-1 panel were underexposed to avoid saturation. Quantification is based on the initial scan. (E) Immunoprecipitates from MCF10A cells expressing vector or shRNA targeting CLCA2 (KD). Endogenous CLCA2 was detected with Sigma Prestige antibody. TD, transduce. TF, transfect. Co-precipitation of CLCA2 with ORAI-1 was detected four times; with STIM-1, six times.

To determine how CLCA2 regulates intracellular calcium, we used immunoprecipitation to examine its interactions with candidate proteins. First we considered Bestrophin 1, which has been shown to modulate SOCE and charging of the ER with calcium by conducting chloride as a counter-ion [**45, 46**]. However, no interaction of CLCA2 was observed with Bestrophin 1 in HEK293 cells (Fig 4A). Therefore we considered the core elements of the calcium sensory and re-charging apparatus.

STIM-1 is a calcium sensor in the ER membrane that interacts and cooperates with the plasma membrane store-operated calcium channel ORAI-1 to modulate ER calcium levels and elicit calcium entry when levels are low [**47**]. We first measured levels of these proteins in HEK293 to rule out the possibility that CLCA2 might simply increase their expression; it did not (Fig 4B). Instead, we found evidence that CLCA2 interacts with both proteins. An antibody recognizing Flag-tagged CLCA2 pulled down 9% of total ORAI-1 from cells expressing CLCA2, and anti-ORAI1 pulled down 11% of total CLCA2 (Fig 4C; see legend for methods). Moreover, both antibodies also pulled down STIM-1. ORAI-1 and STIM-1 are known to form a complex in response to calcium depletion, but in HEK293 cells the complex is easily detected even without calcium-depleting treatments [**48**]. In a separate experiment, anti-STIM-1 also pulled down CLCA2 (Fig 4D). Although the percentage was small, it was observed repeatedly (n = 6). Both the CLCA2 precursor (upper panel) and processing product (lower panel) were precipitated with either protein, suggesting that the interactions were not dependent on cleavage.

These experiments were performed using ectopic expression of CLCA2. To determine whether endogenous CLCA2 interacts with endogenous STIM-1, we used MCF10A cells grown to superconfluency and treated with CPA to activate STIM-1. Immunoprecipitation of CLCA2 pulled down STIM-1, and immunoprecipitation of STIM-1 pulled down CLCA2 (Fig 4E). In these cells, no interaction was observed in the absence of ER calcium depletion by CPA (data not shown).

### 3.5 Cell surface CLCA2 interacts and co-localizes with intracellular STIM-1

**Fig 5:**
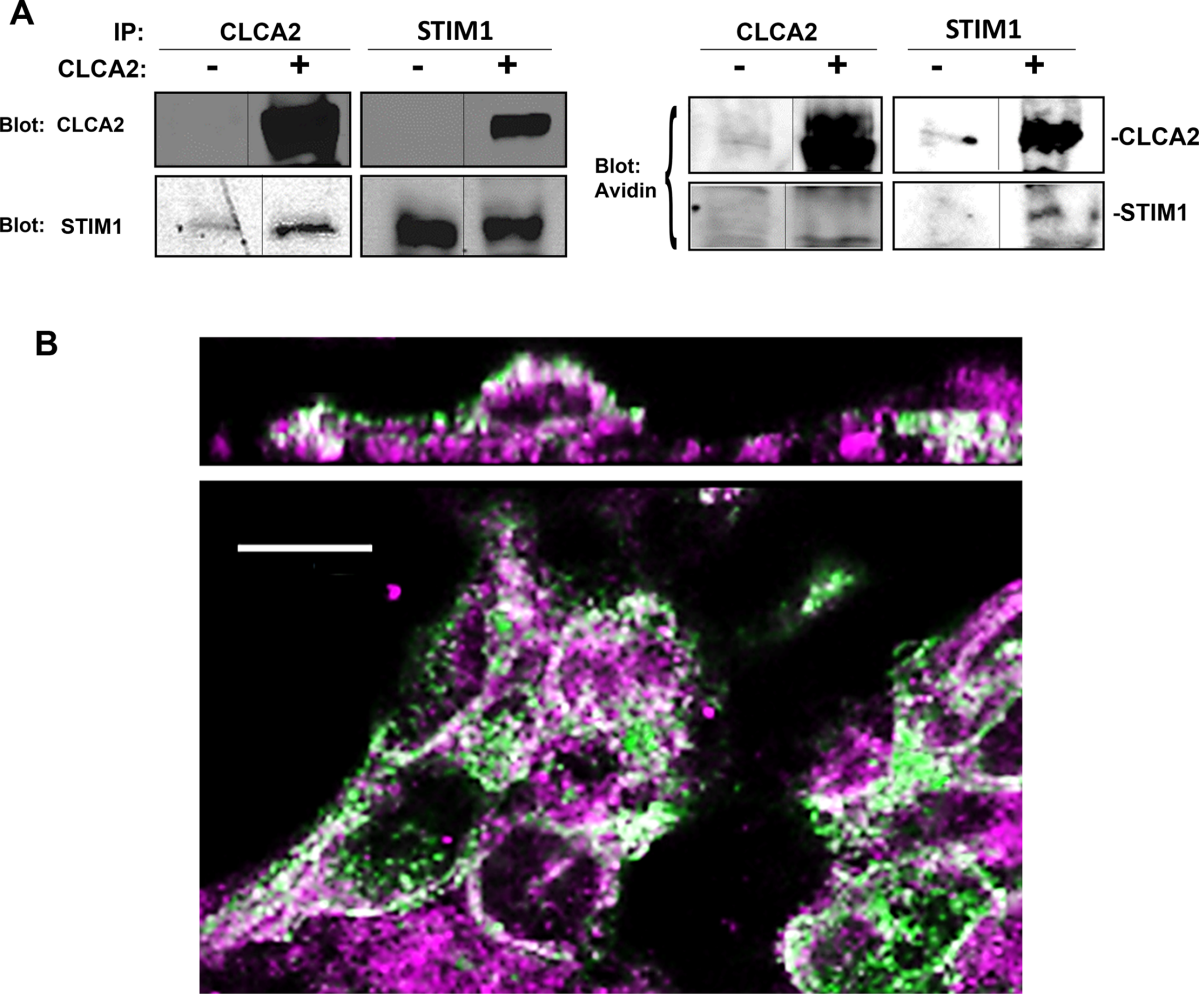
Cell surface CLCA2 co-immunoprecipitates and co-localizes with intracellular STIM-1. (A) Immunoblots of surface-biotinylated cells expressing CLCA2. Biotinylated proteins were detected by neutravidin-Alexa 680. (B) Confocal micrographs of HEK293 cells that stably expressed CLCA2 (green) showed colocalization (white) with STIM-1 (magenta). Top, optical cross-section of *xy* image (bottom). Scale bar, 10 microns.

STIM-1 acts from the ER but may also move to the plasma membrane [**16**]. CLCA2 is a plasma membrane protein but is also found in intracellular vesicles [**49**]. To determine where the proteins interact, we labeled HEK293 cell surface proteins with biotin and performed co-immunoprecipitation. While CLCA2 that co-purified with STIM-1 was biotinylated, STIM-1 that co-purified with CLCA2 was not biotinylated, indicating that surface CLCA2 interacts with intracellular STIM-1, perhaps with ORAI-1 as an intermediary (Fig 5A, right panel).

To determine whether CLCA2 co-localizes with STIM-1, we used immunofluorescence and confocal microscopy on HEK293 cells stably expressing CLCA2. STIM-1 staining (magenta) produced an extensive punctate pattern consistent with ER localization, while CLCA2 (green) occurred both in intracellular vesicles and at cell junctions overlapping (white) with STIM-1. (Fig 5B). An *xz* section (Fig 5B, upper panel) shows CLCA2 at the apical cell surface while STIM1 is intracellular.

### 3.6 CLCA2 enhances conductance of TMEM16A in HEK293 cells

**Fig 6:**
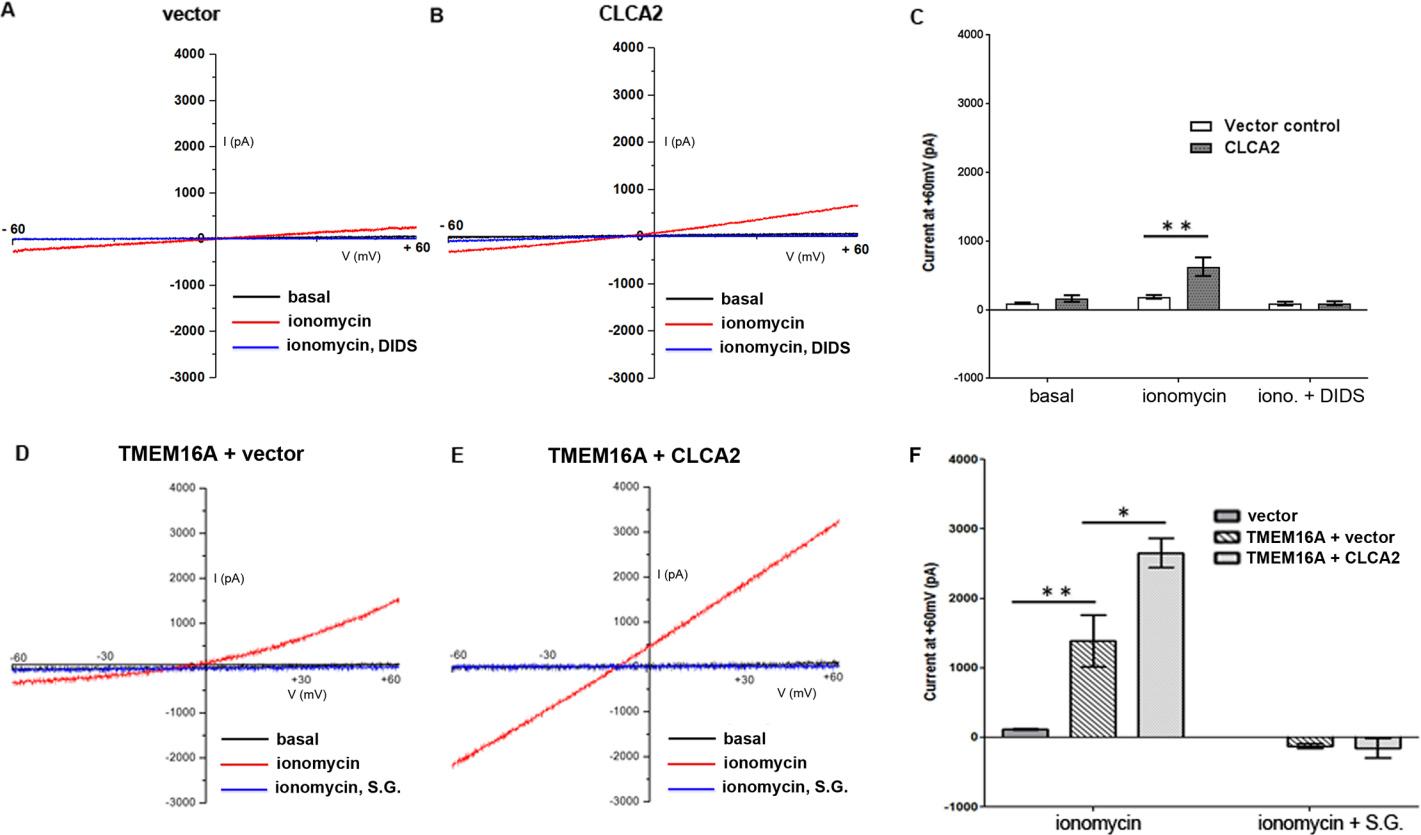
CLCA2 enhances I_ClCa_ in TMEM16A-transduced HEK293 cells. Representative whole-cell chloride currents from cells stably expressing vector (A), CLCA2 (B), TMEM16A plus vector (D) or CLCA2 plus TMEM16A (E) are shown. I_CaCC_ was induced by application of 2 micromolar ionomycin in the presence of extracellular calcium. When the current peaked, a voltage ramp from -60 to +60mV was applied and the current response was recorded. In A and B, current was blocked by DIDS, 100 micromolar. In both D and E, current was fully inhibited by substitution of sodium gluconate (S.G.) for chloride. C, F, relative current amplitudes at +60mV +/- S.E.M. C, n = 9 for CLCA2; n = 7 for vector control; **, p<0.01. F, n = 4 cells for each condition; *, p = 0.02; **, p<0.01. The reversal potential was -8mV in agreement with the Nernst prediction of -7.56mV, based on an excess of 34mM chloride in the extracellular buffer.

To establish the physiological significance of calcium modulation by CLCA2, we investigated its ability to activate I_CaCC_ in HEK293 as reported by Gruber et al. (2000) [**23**]. Cells were transduced with CLCA2, and ionomycin-dependent currents were compared with those of a vector-transduced control. While vector-transfected cells exhibited little current above baseline, cells expressing CLCA2 exhibited dramatically increased current, reaching approximately 600pA at +60mV, that could be inhibited by the chloride channel blocker 4,4'-diisothiocyano-2,2'-stilbenedisulfonic acid (DIDS) (Fig 6A–6C). The reversal potential was -8mV in agreement with the Nernst prediction of -7.56mV, based on an excess of 34mM chloride in the extracellular buffer. The solutions were equiosmolar, ruling out any contribution from volume-regulated chloride channels. These experiments established the baseline I_CaCC_ stimulated by CLCA2 in HEK293.

At least 3 molecules are known to mediate a calcium-activated chloride current, TMEM16A, TMEM16B, and TMEM16F [**4, 7, 50**]. TMEM16A has been reported to mediate this current in HEK293 [**30**]. However, we detected only low levels of TMEM16A mRNA, 0.03+/-0.01% of the level of actin transcript (p<0.05). We were unable to detect any band of the predicted MW, 114kDal, by western blot using an antibody previously reported to detect TMEM16A in HEK293 [**30**]. We concluded that endogenous TMEM16A is present in HEK293 at levels too low to detect immunologically with the tools available. TMEM16B was present at a similarly low level by RT-qPCR. TMEM16F has also been reported in HEK293 [**51**]. These channels could be individually or collectively responsible for the current observed.

Therefore, to establish whether CLCA2 can regulate a specific CaCC, HEK293 cells stably expressing CLCA2 or vector control were transduced with TMEM16A. Cells expressing TMEM16A alone produced a stronger chloride current than cells expressing CLCA2, approximately 1500pA at +60mV, that could be blocked by substitution of gluconate for chloride in the extracellular solution (Fig 6D and 6F). Blockage indicates that the reversal potential had shifted to greater than +60mV, so that no current was detected in this I/V ramp. In cells stably expressing CLCA2 as well as TMEM16A, the chloride current amplitude was almost twofold higher in response to ionomycin, about 2700pA at +60mV (Fig 6E and 6F). This current was also blocked by gluconate substitution, confirming that it was due to chloride. In addition to increased amplitude, the I/V relation became more linear. Others have reported that the I_CaCC_ I/V relation becomes more linear with increasing calcium [**52**]. These results indicated that CLCA2 enhances TMEM16A-mediated chloride current, possibly sensitizing it to calcium.

### 3.7 CLCA2 does not interact with TMEM16A or increase its stability or surface expression

**Fig 7:**
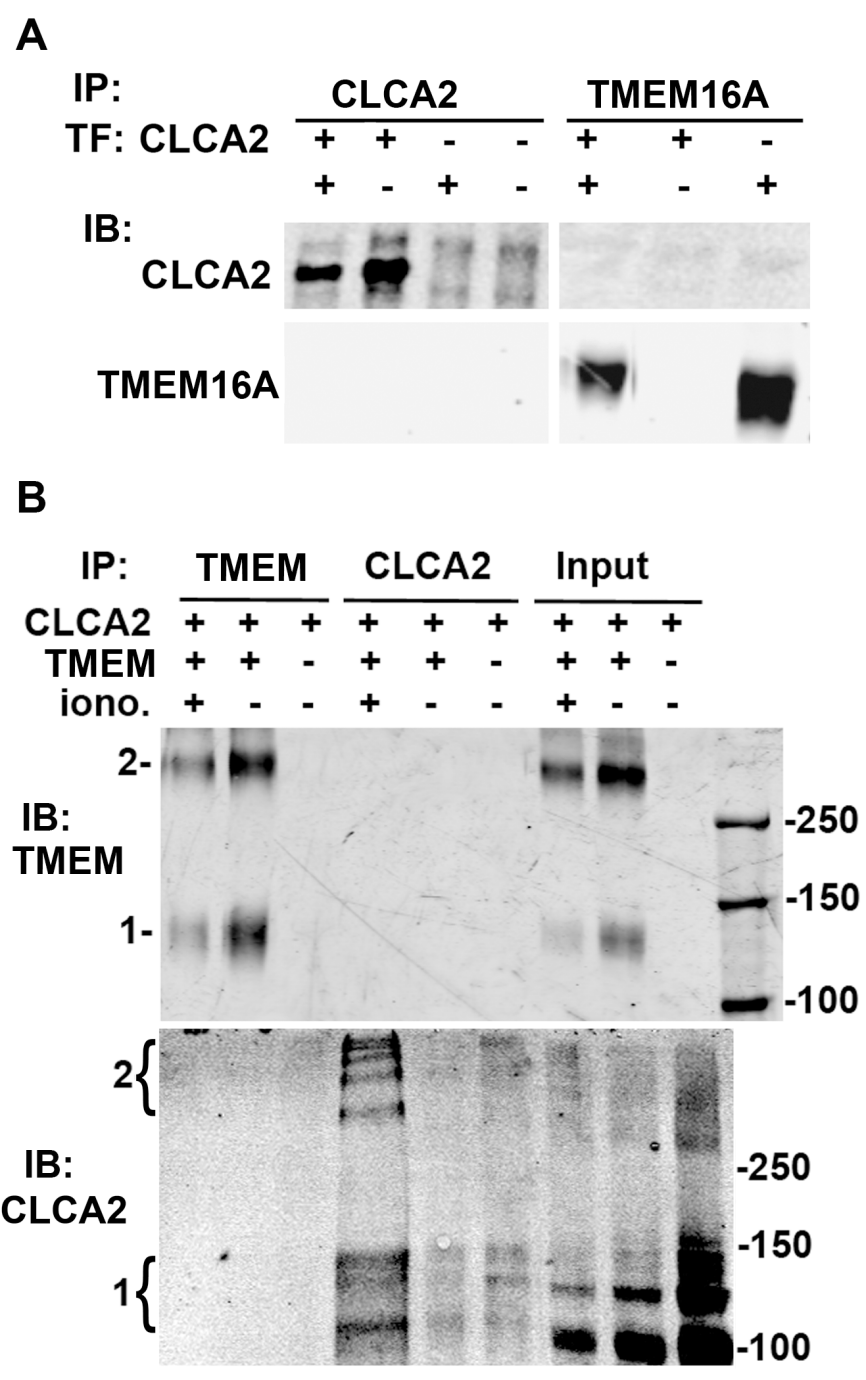
CLCA2 and TMEM16A do not directly interact. (A) Immunoblots of cells transfected with CLCA2, TMEM16A, or both. Proteins were immunoprecipitated and detected with antibodies specific for either protein. (B) Cells stably expressing vector or CLCA2 were transiently transfected with TMEM16A (TMEM) and treated 48h later with the protein cross-linker DSS, followed by immunoprecipitation and immunoblot. TMEM16A was detected with anti-Flag tag antibody, and CLCA2 was detected using TVE20 antibody. In indicated experiments, cells were treated with 1 micromolar ionomycin 5min before adding cross-linker. 1, position of protein monomer. For CLCA2, this includes the 130 kDa precursor and the 100 kDa N-terminal product. 2, apparent multimers. Right, size marker positions are indicated.

CLCA1 has been reported to interact physically with TMEM16A. To test whether CLCA2 likewise interacts with TMEM16A, we performed co-immunoprecipitation (co-IP) and western blot analysis on lysates from HEK293T cells with transient over-expression of CLCA2 and TMEM16A in different concentration ratios. No co-IP was detected (Fig 7A). However, we considered that the interaction might be transient or dependent on the presence of intracellular calcium. Therefore we transfected TMEM16A into HEK293, stably expressing CLCA2 or vector control, and treated cells with the chemical cross-linker DSS in the presence or absence of ionomycin (Fig 7B). Probing immunoblots of cell lysates revealed that both TMEM16A and CLCA2 formed ladders of high-molecular weight bands, suggesting that both proteins exist in multi-protein complexes. However, TMEM16A and CLCA2 were not found to co-IP under any of these circumstances, indicating that there was no physical interaction under the conditions tested.

**Fig 8:**
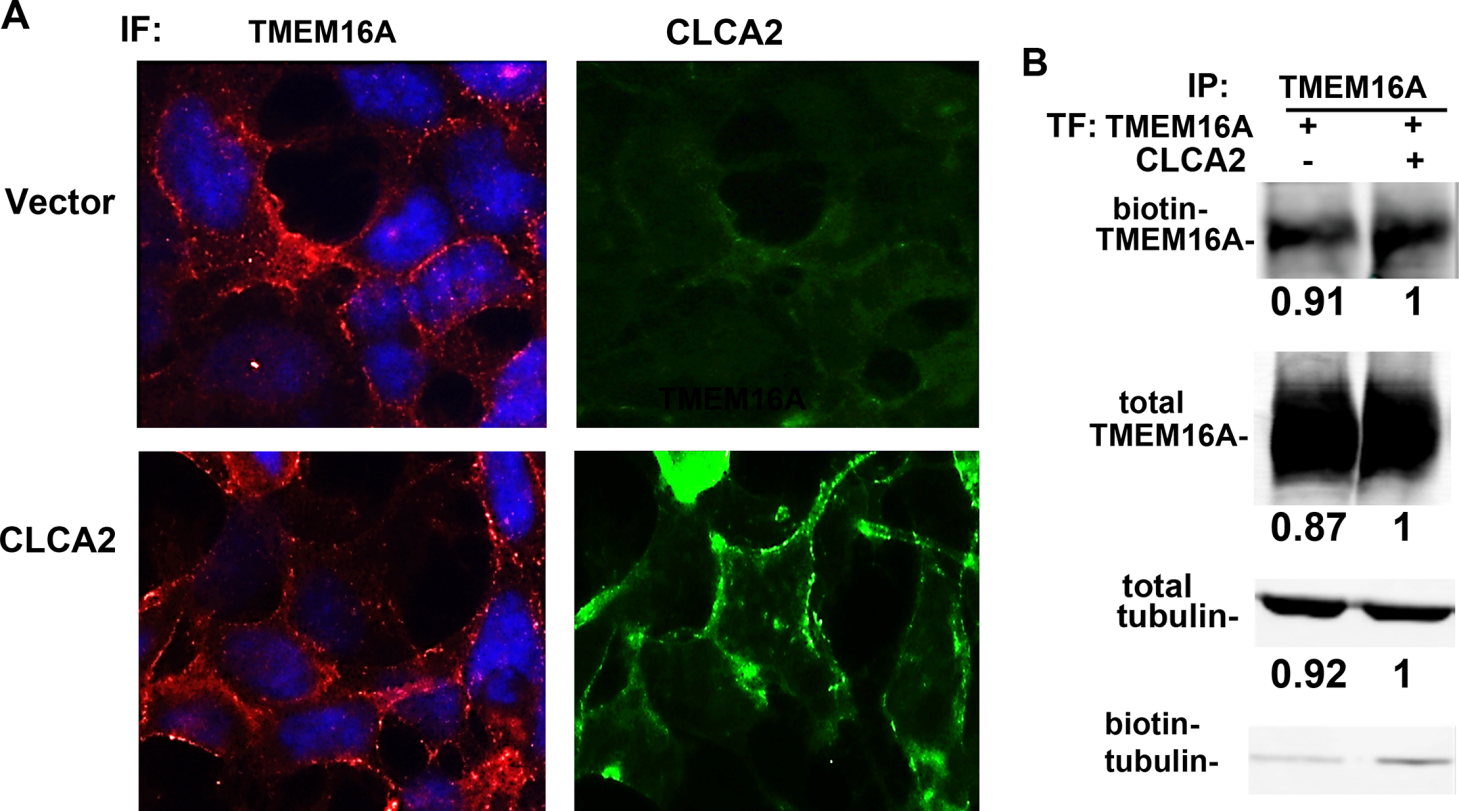
CLCA2 does not increase TMEM16A stability or surface occupancy. (A) Immunofluorescence micrographs showing HEK293 cells stably expressing vector (upper row) or CLCA2 (lower row) and probed for transduced TMEM16A or CLCA2. Cells were fixed and stained with anti-Myc tag antibody for TMEM16A (red) or anti-FLAG antibody for CLCA2 (green). No difference in TMEM16A localization was observed. Scale bar, 20 microns. (B) Immunoblots from surface-biotinylated HEK293 cells stably expressing untagged CLCA2 or vector and transfected with TMEM16A-Flag. TMEM16A was immunoprecipitated, and the blot was probed with Alexa 680-tagged streptavidin. A second blot was probed for TMEM16A. Beta tubulin served as a control for protein concentration. To quantify expression, the fluorescence intensity was determined for each band and normalized to vector control using Licor software. To confirm that biotin labeling was confined to the surface, biotinylated proteins were precipitated with avidin-coated beads, blotted, and probed for beta tubulin, which produced only weak bands.

CLCA1 has been associated with stabilizing TMEM16A at the cell surface [**30**]. To test the same for CLCA2, we first performed immunofluorescence for overexpressed TMEM16A in HEK293 with or without CLCA2. However, the presence of CLCA2 made no qualitative difference in TMEM16A localization (Fig 8A). To measure surface expression biochemically, we used a cell-impermeable biotinylation reagent and performed IP for TMEM16A, then probed with Alexa680-tagged avidin to detect biotinylated surface protein or anti-Flag antibody for total cellular TMEM16A. Expression was quantified by fluorimetric scans and normalized to the vector control. Similar amounts of surface TMEM16A and total TMEM16A were detected regardless of the presence of CLCA2 (Fig 8B). Total and biotinylated tubulin were used to demonstrate that the lysates contained similar amounts of protein and that labeling of intracellular proteins was minimal. Thus, unlike CLCA1, CLCA2 does not affect surface occupancy by TMEM16A.

## 4. Discussion

To gain insight into the conserved function of the CLCA protein family, we sought to identify the mechanism by which CLCA2 regulates calcium-activated chloride channels. We discovered that both CLCA2 and CLCA1 modulate the availability of intracellular calcium. Accordingly, cells expressing CLCA2 and TMEM16A together had approximately twice the chloride conductance of cells expressing TMEM16A alone. These results echoed those of Sala-Rabanal and colleagues [**30**] who studied the effect of CLCA1 on TMEM16A conductance. However, in contrast to their finding that CLCA1 interacted with TMEM16A and stabilized it at the cell surface, we found that CLCA2 did not interact with TMEM16A or affect its stability, dimerization, or surface localization.

Instead, using either a calcium ionophore or a SERCA pump inhibitor to release calcium from the ER, we found that cells expressing CLCA2 had somewhat greater stores of calcium **but much** greater calcium entry when those stores were exhausted, **i.e.**, SOCE. On the other hand, knockdown of CLCA2 in MCF10A reduced both intracellular stores and SOCE. Although several calcium channels have been indirectly linked to TMEM16A activation, it has become clear that TMEM16A is relatively insensitive to global calcium levels and is instead preferentially activated by localized release of calcium from the ER via closely apposed IP3Rs [**3**]. ER calcium filling is regulated by the calcium sensor STIM-1 which gates plasma membrane calcium channels of the ORAI family, especially ORAI-1 in epithelial cells [**47**]. With excessive release of calcium from the ER, STIM-1 dimers anchor to ORAI-1 on the ER-PM junctions, activating ORAI1-mediated calcium influx [**16, 19, 22**]. We discovered that CLCA2 interacts with both STIM-1 and ORAI-1.

But how does direct coupling of CLCA2 to STIM-1 and ORAI-1 result in a robust SOCE response? In principle, CLCA2 might affect the sensitivity of STIM-1 to calcium or regulate the open probability of ORAI-1. On the other hand, since the formation of STIM-1 and ORAI-1 complex is pivotal to SOCE response, CLCA2 might promote complex formation or stabilize it at the plasma membrane. Fig 4C is consistent with the latter. We observed greater co-precipitation of STIM-1 with ORAI-1 in cells expressing CLCA2. Further studies are needed to determine whether this is indeed the primary mechanism of SOCE enhancement. Enhanced SOCE would then lead to increased intracellular calcium stores by SERCA pump activity.

We theorized that regulation of cytosolic calcium might be a conserved feature of the CLCA family and therefore tested cells transduced with CLCA1. We found that CLCA1 had an equally robust effect on calcium stores and SOCE as CLCA2. Thus, it appears that CLCA1 and CLCA2 share one common mechanism for activating TMEM16A but that CLCA1 has acquired an additional capacity to regulate TMEM16A localization, an ability that CLCA2 either lost or never possessed. The relative contribution of the two mechanisms to enhancing chloride conductance remains to be established. It should be noted that Sala-Rabanal et al. [**30**] used different methodology to measure chloride current than employed here. They obviated the role of intracellular calcium by adding calcium to the intracellular buffer, whereas we relied on extracellular calcium admitted by membrane channels. Thus, SOCE could play no role in their studies. CLCA1 and CLCA2 differ significantly in primary sequence, structure, tissue sites of expression, and gene regulation [**27, 28, 53, 54, 55**]. Responding to different cues in different contexts, it is likely that their mobilization of calcium and stimulation of TMEM16A conductance may have different physiological outcomes in different settings. As all CLCA isoforms have been found to enhance I_CaCC_ when transfected into HEK293, it is likely that all amplify cytosolic calcium signaling.

Regulation of TMEM16A itself by CLCA2 has important implications. TMEM16A has a steadily expanding pathophysiological footprint. Its channel activity has been implicated in smooth muscle contraction, transepithelial fluid transport, secretion of mucins and insulin, and, surprisingly, in formation of the primary cilium [**9, 10, 12, 56**]. Probably the best evidence for functional overlap between TMEM16A and the CLCA family is in airway mucin secretion. Both TMEM16A and CLCA1 are associated with IL13-driven expression of Muc5AC. Although CLCA2 is not regulated by IL13, its expression correlates with this mucin in smokers, suggesting some redundancy of function in airways [**57**]. Indeed, deletion of the murine ortholog of CLCA1 can be partly complemented by the ortholog of CLCA2 [**58**]. In another pathophysiological context, TMEM16A plays an important role in vasoconstriction, pulmonary hypertension, and ischemia-induced cardiomyopathy [**13, 59, 60, 61**]. Interestingly, the mouse ortholog of CLCA2 is expressed in aorta, and another homolog is highly expressed in arterial smooth muscle [**62, 63**]. Moreover, a CLCA2 mutation was recently found to cause a familial heart disease, Progressive Cardiac Conduction Defect [**39, 40**].

Independently of its function as a channel, TMEM16A also interacts with EGFR, amplifying its signaling via ERK and CAMKII and consequently enhancing cell proliferation [**64, 65**]. Certain squamous carcinomas and Her2+ breast cancers that are addicted to growth factor signaling amplify TMEM16A to take advantage of this mechanism [**64, 66**]. These cancers often upregulate CLCA2 in tandem [**67, 68**]. Studies are underway to determine whether CLCA2 plays a growth-promoting role in those settings, in contrast to its well established anti-proliferative or anti-migratory role in diverse adenocarcinomas [**34, 35, 37, 69**].

The finding that CLCA proteins regulate cytosolic calcium levels is even more profound. The level of calcium released from the ER is tightly modulated by regulators of proliferation, autophagy, and cell death to suit the physiological circumstances [**70**]. Moderate levels of cytosolic calcium are required for mitochondrial metabolism and cell cycle entry [**71**]. Consequently, some cancers upregulate STIM-1 and ORAI-1 or ORAI3 to ensure a steady supply of low- to mid-level calcium, and cancer cells may become calcium-dependent [**47, 72, 73**]. On the other hand, high-level calcium release from the ER to mitochondria via IP3Rs promotes apoptosis [**70**]. Thus, IP3R is tightly regulated by pro-survival proteins of the Bcl2 family and by AKT1 phosphorylation [**70, 74**]. In response to cytotoxic stress such as DNA damage, BRCA1 enhances the open probability of IP3R, and p53 activates SERCA pumps and promotes ER-mitochondrial membrane association; these effects enhance calcium release to mitochondria, and lead to loss of membrane potential, release of cytochome C, and activation of the intrinsic apoptotic cascade [**75**]. The latter is important because CLCA2 is highly induced by p53 in response to DNA damage, and overexpression of CLCA2 is sufficient to induce apoptosis in some cell types [**35**]. It is possible that p53 employs CLCA2 in severely damaged cells to promote cell death by amplifying SOCE.

Calcium also plays a role in epithelial differentiation, by promoting the maturation of cell-cell junctional structures and anchoring them to the cytoskeleton [**15**]. CLCA1 and CLCA2 have been shown to promote differentiation of distinct epithelial cell types [**36, 76**]. In mammary epithelial cells, CLCA2 interacts with homophilic cell-cell adhesion molecule EVA1, and its cytoplasmic tail binds the regulatory molecules beta catenin and ZO-1 [**49**]. Loss of CLCA2 triggers epithelial-to-mesenchymal transition in mammary epithelial cells [**36**]. In other contexts, ligation of EVA1 has been shown to trigger release of intracellular calcium [**77**]. Based on the results presented here, it is tempting to speculate that CLCA2 promotes this and other calcium-mediated events in differentiation.

In summary, our discovery that both CLCA2 and CLCA1 modulate intracellular calcium levels suggests that this may be the common function of the family. Future studies exploring the role of CLCA proteins in the many physiological processes regulated by calcium signaling may open new therapeutic avenues.

## Abbreviations

AKT-1: Rac-alpha Serine/Threonine Protein Kinase-1
TMEM16A: Anoctamin1
ATP: Adenosine Tri-Phosphate
BCA: Bicinchoninic acid
Bcl-2: B-cell lymphoma-2
BRCA-1: Breast Cancer gene-1
BSA: Bovine Serum Albumin
BTP-2: N-[4-(3,5-Bis(trifluoromethyl)-1H-pyrazol-1-yl]phenyl]-4-methyl-1,2,3-thiadiazole-5-carboxamide
CAMKII: Calcium/Calmodulin-dependent Kinase II
CaCC: Calcium-activated Chloride Channel
CLCA: Chloride Channel Accessory
CPA: Cyclopiazonic Acid
DMSO: Dimethyl sulfoxide
DSS: Disuccinimidyl suberate
DTT: Dithiothreitol
EGFR: Epidermal Growth Factor Receptor
EGTA: Ethylene glycol-bis(β-aminoethyl ether)-N,N,N′, N′-tetraacetic acid
ER: Endoplasmic reticulum
ERK: Extracellular signal-Regulated Kinase
HEK: Human Embryonic Kidney
HEPES: 4-(2-hydroxyethyl)-1-piperazineethanesulfonic acid
IHC: Immunohistochemistry
I_CaCC_: calcium-activated chloride current
IP3: Inositol triphosphate
IP3R: Inositol Triphosphate Receptor
MCF10A: Michigan Cancer Foundation-10A
PLC: Phospholipase C
SERCA: Sarcoendoplasmic Reticulum Calcium-ATPase transporter
shRNA: Short-hairpin Ribonucleic Acid
SOCE: Store-Operated Calcium Entry
STIM-1: Stromal Interaction Molecule 1
ORAI-1: Calcium Release Activated Calcium Modulator-1
ZO-1: Zonula Occludens-1
